# Practices in reporting incidental findings in lung cancer screening by low-dose CT: a European Survey of Radiologists by SOLACE

**DOI:** 10.1186/s13244-026-02257-w

**Published:** 2026-05-29

**Authors:** Roberta Eufrasia Ledda, Emily Nischwitz, Hans-Ulrich Kauczor, Marie-Pierre Revel, Eva Kočová, Jens Vogel-Clausen, Torsten Gerriet Blum, Joanna Chorostowska-Wynimko, Helmut Prosch, Anna Kerpel-Fronius, SOLACE Collaborative Authors, Helmut Prosch, Marie-Pierre Revel, Monika Hierath, Coline Mathonier, Katarzyna Błasińska, Jens Vogel-Claussen, Kyriaki Tavernaraki, Iris Vlachantoni, Anna Kerpel-Fronius, Ildikó Horváth, Steven Schalekamp, Nuala Healy, James Ryan, Istvan Levente Kui, Eva Kočová, Martina Vašáková, Lucija Kovačević, Maja Prutki, Viktoria Palm, Oyunaa von Stackelberg, Elizabeth Wai Yee Tong, Mathis Konrad, Guillermo Gallardo, Roberta Eufrasia Ledda, Gianluca Milanese, Pilvi Ilves

**Affiliations:** 1 Department of Medicine and Surgery, University of Parma, Parma, Italy; 2 Fondazione IRCCS Istituto Nazionale dei Tumori, Milan, Italy; 3 Clinic for Diagnostic and Interventional Radiology (DIR), Heidelberg University Hospital, Heidelberg, Germany; 4 Translational Lung Research Center Heidelberg (TLRC), German Center for Lung Research (DZL), Heidelberg, Germany; 5 Diagnostic and Interventional Radiology with Nuclear Medicine, Thoraxklinik Heidelberg, Heidelberg, Germany; 6 Department of Radiology, Hôpital Cochin, AP-HP, Paris, France; 7 Faculté de Médecine, Université Paris Cité, Paris, France; 8 Fakultni Thomayerova Nemocine (TN), Prague, Czechia; 9 Institute for Diagnostic and Interventional Radiology, Hannover Medical School, Hannover, Germany; 10 Biomedical Research in Endstage and Obstructive Lung Disease Hannover (BREATH), German Center for Lung Research (DZL), Giessen, Germany; 11 Medical School Berlin, Berlin, Germany; 12 Lungenklinik Heckeshorn, Helios Klinikum Emil von Behring, Berlin, Germany; 13 Department of Genetics and Clinical Immunology, National Institute of Tuberculosis and Lung Diseases, Warsaw, Poland; 14 Department of Biomedical Imaging and Image-Guided Therapy, Medical University of Vienna, Vienna, Austria; 15 National Koranyi Institute for Pulmonology, Budapest, Hungary; 16 Assistance Publique Hopitaux de Paris (APHP), Paris, France; 17 EIBIR Gemeinnutzige Gmbh Zur Forderung Der Erforschung Der Biomedizinischen Bildgebung, Vienna, Austria; 18 IInstytut Gruzlicy i Chorob Pluc (IGICHP), Warsaw, Poland; 19 Medizinische Hochschule Hannover, (MHH), Hannover, Germany; 20 Hellenic Ministry of Health (MoHGR), Athens, Greece; 21 Orszagos Koranyi Pulmonologiai Intezet (NKIP), Budapest, Hungary; 22 Radboud Universitair Medisch Centrum (RADBOUDMC), Nijmegen, the Netherlands; 23 The Royal College of Surgeons in Ireland (RCSI), Dublin, Ireland; 24 Szabolcs-Szatmár-Bereg Vármegyei Oktatókórház (SZSZBVK), Nyiregyhaza, Hungary; 25 Fakultní Thomayerova Nemocnice (TN), Prague, Czech Republic; 26 Klinički Bolnički Centar Zagreb, Zagreb, Croatia; 27 Universitatsklinikum Heidelberg (UKHD), Heidelberg, Germany; 28 Universidad de Navarra (UNAV), Pamplona, Spain; 29 Universita Degli Studi di Parma (UNIPR), Parma, Italy; 30 Tartu Ülikool (UTARTU), Tartu, Estonia

**Keywords:** Lung cancer screening, Incidental findings, Survey

## Abstract

**Objective:**

Lung cancer screening (LCS) is complicated by a high prevalence of incidental findings (IFs), defined as abnormalities detected on low-dose computed tomography (LDCT) of the chest that are outside the scope of LCS. Although the detection of some abnormalities might lead to the diagnosis of conditions of clinical relevance, LDCT protocols are not adequate for the characterisation of most IFs and thus, further investigations are usually necessary. Thus, the detection of such findings is perceived as a double-edged sword, and radiologists still widely disagree on which IFs should be reported.

**Materials and methods:**

A survey was circulated amongst radiologists involved in LCS programmes across Europe to investigate their opinions regarding the reporting of IFs, based on their personal experiences and perspectives, as well as national regulations. The responses of 147 European radiologists were included in the final analysis.

**Results:**

The survey revealed a lack of standardised regulations and limited awareness among European radiologists regarding IFs reporting in LCS. LDCT is perceived as unreliable for evaluating solid organs outside the mediastinum, and mandatory reporting is supported only for clinically relevant findings.

**Conclusion:**

With radiologists still partly disagreeing on which IFs should be reported, international evidence-based guidelines around IFs reporting and management are highly awaited but challenging.

**Critical relevance statement:**

This study critically highlights the lack of standardised regulations and consensus on IFs reporting in European LCS, underscoring the urgent need for harmonised, evidence-based guidelines to advance consistency and quality in clinical radiology practice.

**Key Point:**

- European radiologists report a lack of standardised regulations and awareness regarding IFs in LCS.
- LDCT is widely perceived as unreliable for assessing solid organs outside the mediastinum, limiting support for mandatory reporting of extra-thoracic findings.
- International, evidence-based guidelines are urgently needed to harmonise IFs reporting and management in LCS across Europe.

**Graphical Abstract:**

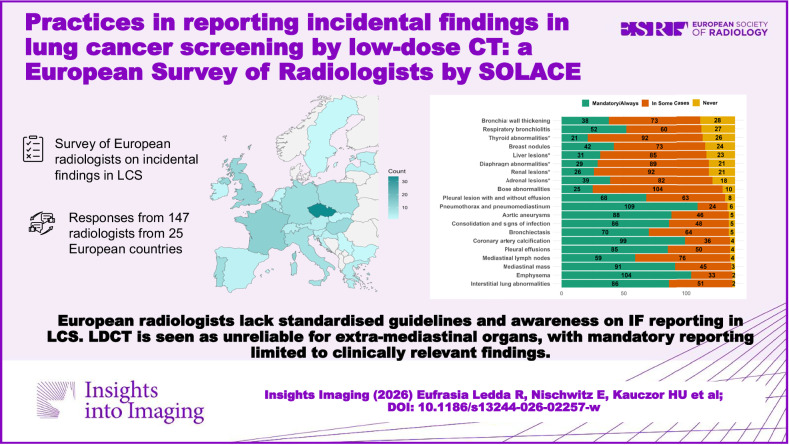

## Introduction

With the progressive expansion of lung cancer screening (LCS) programmes across Europe [1], handling the issues related to reporting and managing incidental findings (IFs) has become mandatory. IFs are broadly defined as abnormalities detected on low-dose computed tomography (LDCT) that are outside the primary scope of LCS [2], which remains the diagnosis of lung cancer (LC) at an early stage. There are some lines of evidence that demonstrate that some IFs are associated with increased mortality [3–5], underlining the importance of standardised reporting to ensure consistent evaluation and management [6]. Different classifications have been proposed for these commonly detected findings [7]; one is based on location, whereby IFs are categorised into thoracic and extra-thoracic. However, some thoracic findings, such as coronary artery calcification (CAC) and pulmonary emphysema, which are expected in heavy smokers (either ex- or current), are considered more as comorbidities and thus, defined as “additional” findings rather than “incidental” [8]. A more practical classification distinguishes clinically significant from insignificant findings [9], but providing an unequivocal definition of what is clinically significant is rather challenging, as well as of what is clinically “actionable” from “non-actionable”.

Regardless of the limitations related to both the definition and classification of IFs in LCS, their reporting is a double-edged sword. The detection of some abnormalities might allow for the diagnosis of serious conditions, with the potential of improving overall outcomes, but LDCT protocols are not adequate for the characterisation of all IFs, and thus, further diagnostic work-up might be recommended. Such a scenario can lead to additional tests and procedures, including invasive ones, that may be futile or even harmful, increasing the risk of overdiagnosis and overtreatment of IF [10].

To date, there is still a lack of scientific evidence regarding the predictive and prognostic impact of reporting IFs in LCS, as well as the derived preventive or therapeutic implications, likewise the impact on the cost-effectiveness of LCS programmes [9, 11, 12]. International guidelines on LCS lack precise details on IFs reporting and management, and radiologists widely disagree on which IFs could be reliably detected in LDCT or should be reported [13]. Based on currently available evidence, here is a consensus-based official statement of six leading European professional societies for reporting and management of IFs [2]. However, another official Statement of the American Thoracic Society (ATS) identifies the knowledge gaps and emphasises the need for further research on reporting and management of IFs detected on LDCT, especially CAC [14].

The aim of this survey study was to investigate the opinions of radiologists involved in LCS programmes across Europe regarding the reporting of IFs, based on their personal experiences and perspectives.

## Materials and methods

A team of experts from the EU4Health-funded SOLACE project (Strengthening the screening of Lung Cancer in Europe) reviewed and discussed how to best assess the current regulations and opinions surrounding IFs in LCS. This group consisted of radiologists and pulmonologists from actively recruiting SOLACE LCS sites. Details on the SOLACE project can be found elsewhere [15]. Survey data were collected and managed using REDCap electronic data capture tools hosted at University Hospital Heidelberg (UKHD) [16, 17].

This survey was active from October 28, 2024, to April 1, 2025, and the link to the survey was disseminated through email to the SOLACE LCS sites and partners, the European Society of Thoracic Imaging (ESTI) contact list, and several national thoracic radiology mailing lists.

Ethics committee approval was waived because the study was based on an anonymous, voluntary survey addressed to healthcare professionals and did not involve patients, patient data, or any identifiable personal information.

The survey targeted radiologists, respondents from other professions were excluded through an initial screening question. Participants were asked to indicate the country in which they conduct the majority of their LCS reporting. Responses from professionals outside of Europe (*n* = 3) were removed from the final analysis.

Three multiple-choice questions were asked

1. The type of screening programmes the respondent is involved in (national, research/implementation pilot, not within a programme, other).
2. The levels at which LCS reporting is regulated (national, regional, research pilot, not regulated).
3. The levels at which IFs are part of LCS reporting are regulated (national, regional, research pilot, and not regulated).

If any level of regulation for IFs as part of LCS reporting was indicated, respondents were further asked to indicate how each finding is regulated (mandatory/always, in some cases, never, or not regulated). The 20 findings assessed included: CAC, interstitial lung abnormalities (ILAs), pulmonary emphysema, bronchiectasis, consolidation and sites of infection, bronchial wall thickening, mediastinal lymph nodes, thyroid abnormalities, mediastinal mass, pleural effusions, pneumothorax and pneumomediastinum, diaphragm abnormalities, aortic aneurysms, breast nodules, liver lesions, renal lesions, bone abnormalities, adrenal lesions, respiratory bronchiolitis, and pleural lesion with and without effusion.

All participants were then asked to rank their level of agreement with the following statement: LDCT is not a reliable technique to assess solid organs outside of the mediastinum. If this and the preceding questions were not answered, the respondent was removed due to insufficient information provided.

Then, participants were asked to give their opinion on how they thought each of the above findings should be regulated in the reporting (mandatory/always, in some cases, never). For each finding/abnormality, which in some cases was indicated, a follow-up question was asked to give their opinion on what degree of severity each condition should be regulated in the reporting of IF (high severity, moderate and high severity, or low, moderate, and high severity).

The full survey can be found in Supplemental Fig. 1.

## Results

### Demographic and geographic characteristics of survey respondents

The responses from 147 individuals were included in the final analysis of the survey, comprising 134 complete and 13 partial responses. Partial responses were defined as those in which participants completed the demographic and regulation-related questions but did not complete all opinion-based questions.

**Fig. 1:**
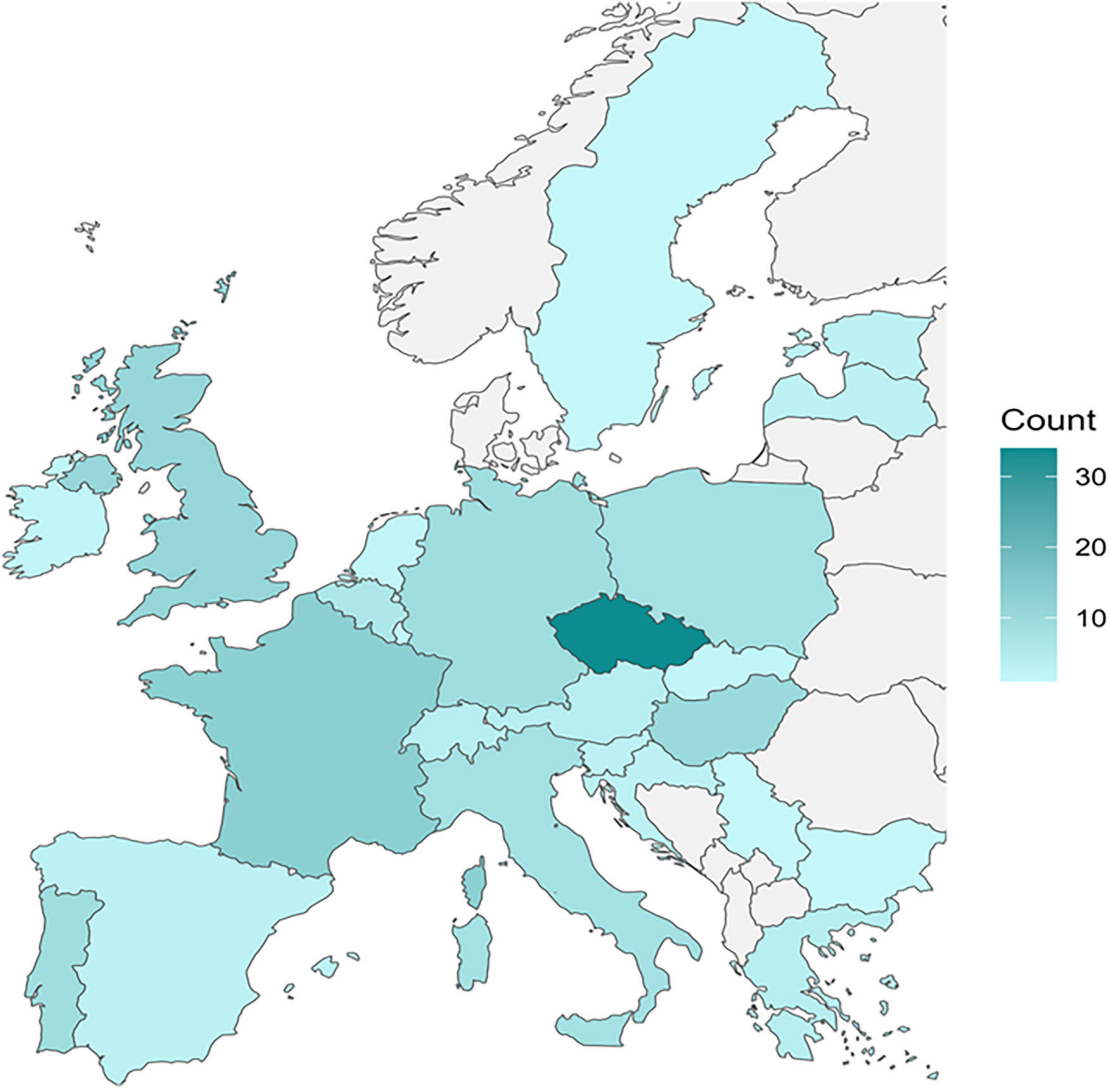
Overview of survey responses by country. Countries are shaded in teal according to the distribution of responses, with the lighter shade indicating those with fewer responses (minimum: *n* = 1) and the darker shade indicating more responses (maximum: *n* = 34). Countries with no response are shown in grey

The survey captured responses from individuals from 25 European countries. Czechia was the most represented (*n* = 34, 23.1%), followed by France (*n* = 13, 8.8%) and the United Kingdom (*n* = 12, 7.5%) (Fig. 1 and Supplemental Table 1). The LCS programme types in which the respondents were involved were research and implementation (*n* = 68, 41.0%), non-programmatic (*n* = 44, 26.5%), national (*n* = 47, 28.3%), and other (*n* = 7, 4.2%).

### Regulatory requirements for LCS reporting and IFs

**Fig. 2:**
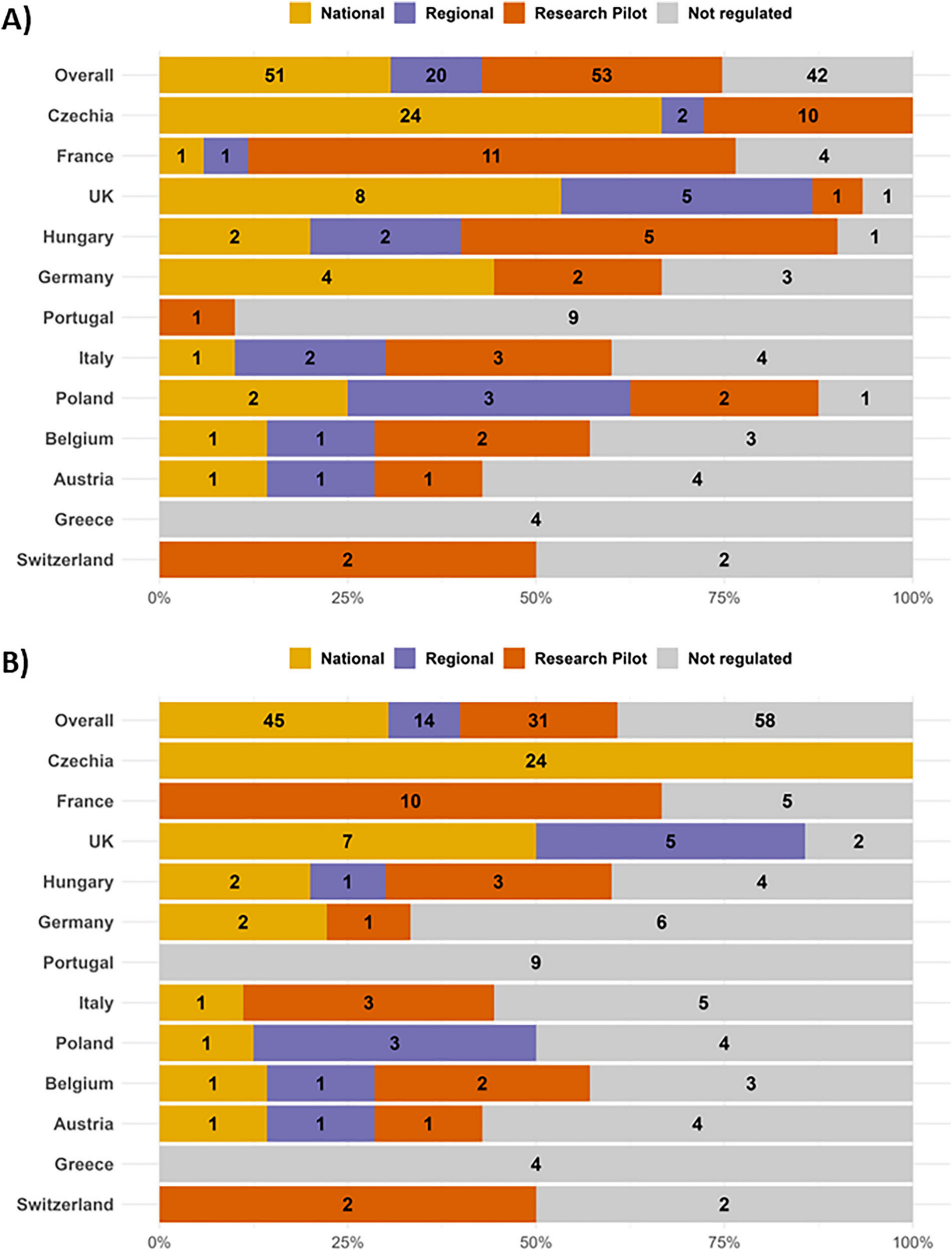
Radiologist-reported regulatory frameworks for LCS and IF reporting. Summary of respondents’ perceptions of how LCS (**A**) and IF (**B**) reporting are being regulated in their countries, at the national level (yellow), regional level (purple), through research pilot protocols (orange), or not regulated (grey). Only countries with *n* ≥ 4 survey responses are shown. The Overall bar aggregates all submitted responses across all countries (*n* = 166)

To assess radiologists’ familiarity of the regulatory landscape surrounding LCS reporting, participants were asked to indicate all applicable levels of regulation which have oversight to both LCS reporting (Fig. 2A) and IF reporting (Fig. 2B). Namely, the survey questions were: “At which level(s) is lung cancer screening reporting regulated?” and “At which level(s) is incidental finding as part of lung cancer screening reporting regulated?”. Possible levels of regulation were national, regional, research pilot protocol, or not regulated. The responses revealed heterogeneity, with little consensus across countries. Among countries with more than three respondents (*n* = 17), only three and four countries indicated completely consistent responses for LCS reporting and IFs reporting, respectively. Thus, highlighting the lack of familiarity of respondents with the current regulations of LCS and IFs reporting.

**Fig. 3:**
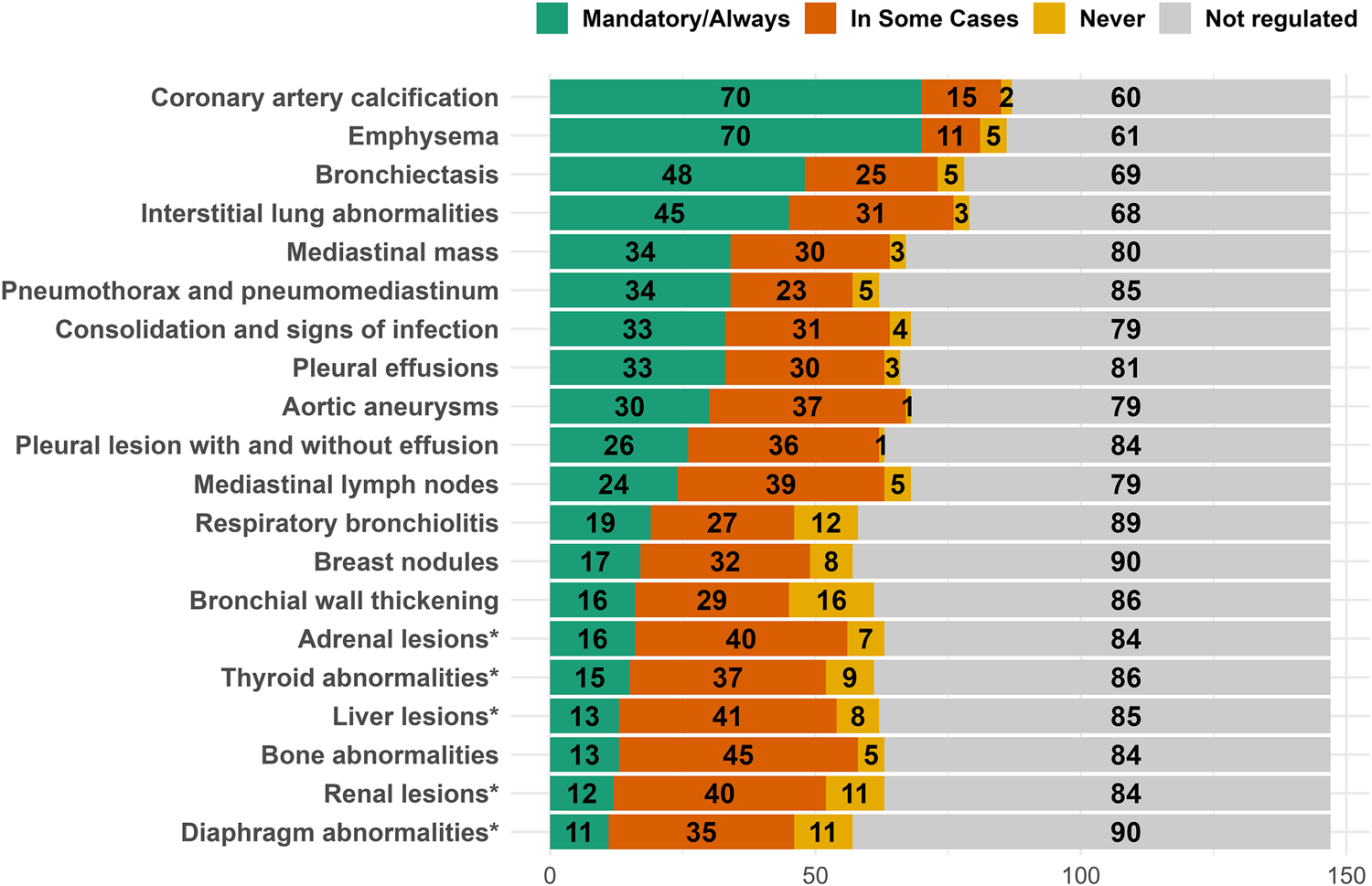
Radiologist-reported regulatory frameworks for IF reporting. Summary of respondents’ perceptions of how conditions in LCS reporting are being regulated in their countries, mandatory/always (green), in some cases (orange), never (yellow), and not regulated (grey). *Indicate extra-thoracic conditions (*n* = 147)

Radiologists who indicated any level of regulation for IFs reporting were subsequently prompted to specify how each of the 20 pre-selected findings is regulated, categorised as mandatory/always, in some cases, never, or not regulated (Fig. 3). For most findings (16 of 20), over 50% of responses indicate that they are not regulated. Only two findings, CAC and emphysema, had more than 50% combined responses of mandatory/always and in some cases. Notably, the five extra-thoracic findings indicated with an asterisk (adrenal lesions, thyroid abnormalities, liver lesions, renal lesions, and diaphragm abnormalities) ranked among the lowest in both mandatory/always and in some cases responses. Fewer than 10.8% (*n* = 16) of respondents indicated mandatory/always reporting regulation for any of these findings.

### Opinions surrounding the reporting of IFs

**Fig. 4:**
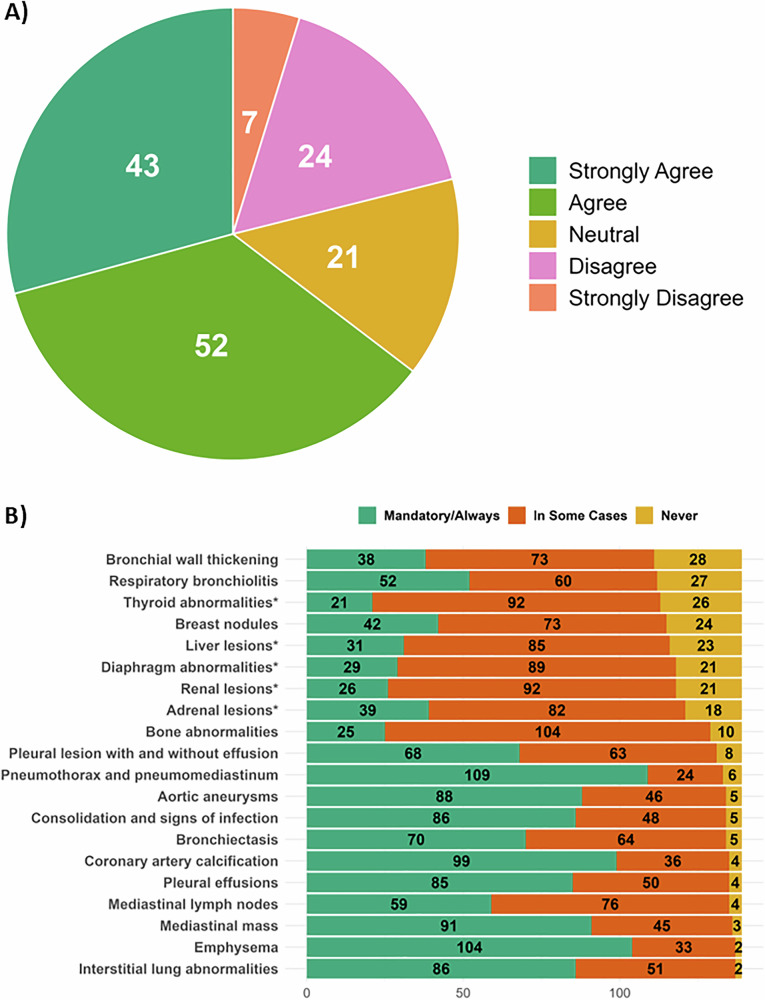
Opinions from Radiologists on the Reporting of IFs. **A** Pie chart illustrating the degree of agreement with the statement “LDCT is not a reliable technique to assess solid organs outside of the mediastinum.” **B** Bar plot summarising the opinions of radiologists on how various conditions should be conditionally reported: mandatory/always regardless of severity (green), in some cases, dependent on severity (orange), and never reported (yellow) (*n* = 139)

All participants (*n* = 147) rated their level of agreement or disagreement with the following statement: “LDCT is not a reliable technique to assess solid organs outside of the mediastinum” (Fig. 4A). Respondents either strongly agreed (*n* = 43, 29%), agreed (*n* = 52, 35%), neither agreed or disagreed (*n* = 21, 14%), disagreed (*n* = 24, 16%), or strong disagreed (*n* = 7, 5%) with this statement.

To obtain more finding-specific opinions, participants were also asked if, in their opinion, if each of the 20 findings should be reported on a mandatory/always basis (independent on severity), in some cases (dependent on severity), or never reported (Fig. 4B). The survey question was: “Indicate how each condition in your opinion should be regulated in the reporting of incidental findings:”. Respondents (*n* = 139) indicated in over 70% that a pneumothorax/pneumomediastinum (*n* = 109, 78.4%), emphysema (*n* = 104, 74.8%), and CAC (*n* = 99, 71.2%) should be mandatorily reported. The five-extra thoracic findings were amongst the top conditions suggested to never be reported or only to be reported in some cases (*n* = 18–26, 18.7–12.9%).

## Discussion

This survey assessed European radiologists’ familiarity with the regulatory landscape surrounding LCS reporting, including IFs, and captured their opinions about IFs reporting in the setting of LCS.

As expected, the responses obtained first demonstrated the absence of standardised reporting regulations across Europe. Indeed, in Europe, there are only four countries which have national-level guidelines on LCS. In Croatia, in January 2020, the LCS national guidelines were issued by the Croatian Ministry of Health in cooperation with the Croatian Thoracic Society and the Croatian Society of Radiology [18]. In Czechia, the guidelines were produced within the national LCS programme and then approved and finalised by the Czech Ministry of Health, the Czech Radiologic Society, and Nuclear Safety Regulators in February 2021 [19]. In September 2022, the UK National Screening Committee recommended introducing targeted LCS, and standard protocols and quality assurance standards for the LCS Programme were initially published in February 2019, with a recent update in March 2025, by the National Health Service [20–22]. In June 2024, the German Federal Ministry for the Environment, Nature Conservation, Nuclear Safety and Consumer Protection (BMUV) issued a statement allowing for the use of LDCT for LCS [23]. Shortly following this ordinance, in June 2025, the Federal Joint Committee (G-BA) passed the resolution to support the funding of LCS through statutory health insurance providers [24]. In April 2025, the S3 Guideline on Prevention, Diagnosis, Therapy, and Follow-up of Lung Cancer was published, including specifics on LCS [25].

When assessing radiologists’ awareness about the existing regulations around LCS and IFs reporting, a high variability of responses was registered, even among radiologists from the same country. Notably, participants from only four countries provided consistent responses on IFs reporting. Moreover, among those IFs for which the existence of a certain level of regulation was reported, only CAC and pulmonary emphysema had more than 50% combined responses of mandatory/always and in some cases. Such relatively higher percentages were somehow expected, likely based on the evidence that pulmonary emphysema is independently associated with increased LC incidence and mortality, as well as with higher rates of all-cause and respiratory disease-related mortality [26, 27]. Similarly, some LCS trials have demonstrated a positive correlation between CAC and cardiovascular-related mortality [4, 5] and between high CAC burden and all-cause mortality [28–30]. ILA had nearly half of the responses of mandatory/always, suggesting a suboptimal level of awareness among respondents regarding their potential clinical significance. Indeed, according to the recently issued ATS Clinical Statement on ILAs, the presence or absence of such findings in LCS participants should be systematically assessed and reported [31]. This recommendation relies upon large evidence demonstrating that subjects with ILAs are at increased risk for respiratory symptoms, impaired lung function, and all-cause mortality [32]. The prevalence of ILAs is thought to be significantly higher in LCS participants than in the general population. More recent evidence, however, showed slightly higher prevalence in LCS (7.1 vs 6.8%) [33]. Approximately 50% of cases show radiological progression, with a higher rate of progression over a longer follow-up period and in those subjects displaying fibrotic ILAs [32, 33]. Conversely, extra-thoracic findings along with breast nodules and bone abnormalities – not strictly extra-thoracic in location—ranked among the lowest in both mandatory/always and in some cases responses. These results were in keeping with those observed in the second part of the survey that aimed at gathering respondents’ personal opinions around IFs reporting, whereby almost 65% of participants agreed that chest LDCT is not a reliable technique for assessing solid organs outside of the mediastinum. Of note, most respondents indicated that extra-thoracic findings, along with breast nodules and bone abnormalities, should be either not reported or reported in some cases, whereas CAC, pulmonary emphysema and IFs of potential clinical significance (i.e., pneumothorax, pneumomediastinum, and aortic aneurysm) should be either mandatory/always or in some cases. As already mentioned, providing a clear and unequivocal definition of what is “clinically significant” is rather challenging, as partly subjective to personal interpretation. This is why an alternative proposed classification distinguishes “actionable” from “not actionable” IFs, with the former being represented by those abnormalities needing further evaluation to clarify their nature. However, the concept of “actionable” is also prone to subjective interpretation [6]. Nonetheless, we did not ask respondents to indicate which findings they thought were either clinically significant/not clinically significant or actionable/not actionable.

Overall, the obtained responses seem to reinforce the scepticism around the routine reporting of IFs in LCS, and particularly that of extra-thoracic ones. The reasons for such scepticism can be found in the well-recognised drawbacks of LCS [6], including the risk of overcalling and overdiagnosis, increasing financial costs, increasing radiologists’ workload and the potential psychological burden on participants. While the potential risk of overcalling/overdiagnosis is intrinsic to every type of screening programme, there are continuous efforts to improve both the radiological and clinical management of both lung nodules and IFs. To date, there are very few guidelines available on follow-up of IFs, especially those with potentially limited clinical significance despite their prevalence (up to 90% according to some studies) [34, 35]. The detection and subsequent reporting of abnormalities that would not have caused symptoms or affected a person’s lifespan if left untreated can lead to a cascade of additional tests, having a negative impact on radiologists’ workload, who will have more imaging investigations to perform and report, and on healthcare financial resources [11, 36]. Based on the evidence that nearly half of the per-patient reimbursement for LCS was related to management of IFs, Morgan et al highlighted the need for each screening programme to assess the optimal means of managing IFs based on the available economic resources [34]. Regardless of the detection of IFs, LCS is well-known to cause short-term increased anxiety [37]. Nonetheless, the level of anxiety of a given LCS participant is likely to increase even more when informed of the presence of further abnormalities besides pulmonary nodules. Therefore, the topic of appropriate communication of the IFs needs further attention and training for LCS personnel.

This survey has some limitations. First, the number of subjects recruited to participate was rather small, and not all questions were answered by all participants. Moreover, the 25 European countries were not uniformly represented among the respondents, with a particular bias towards Czechia. Also, the survey did not evaluate the extent of the respondents’ radiological experience, which could have a potential impact on the results. Finally, some IFs were not included in the analysis (e.g., heart valve calcifications).

In conclusion, our survey highlighted the absence of standardised reporting regulations in LCS across Europe, and the lack of radiologists’ awareness about the existing regulations surrounding IFs reporting. The results also showed that most radiologists agreed that LDCT protocols are not adequate for the assessment of solid organs outside of the mediastinum, confirming a general scepticism around the reporting of extra-thoracic IFs in LCS. With radiologists still partly disagreeing on which IFs should be reported, international evidence-based guidelines around IFs reporting and management are highly awaited but challenging.

## Supplementary information

ELECTRONIC SUPPLEMENTARY MATERIAL

## Data Availability

Data available upon request.

## Abbreviations

CAC: Coronary artery calcification
IFs: Incidental findings
ILAs: Interstitial lung abnormalities
LDCT: Low-dose computed tomography
LC: Lung cancer
LCS: Lung cancer screening
SOLACE project: Strengthening the screening of Lung Cancer in Europe
