# Supplementary material for: Practices in reporting incidental findings in lung cancer screening by low-dose CT: a European Survey of Radiologists by SOLACE
Source: Insights Imaging. 2026 May 29;17:147. doi: 10.1186/s13244-026-02257-w (PMC13221548; doi:10.1186/s13244-026-02257-w)
Supplement: Supplementary file 1 — ELECTRONIC SUPPLEMENTARY MATERIAL [file 13244_2026_2257_MOESM1_ESM.pdf]

Practices in Reporting Incidental Findings in Lung Cancer Screening by Low-dose CT: A European Survey of Radiologists by SOLACE

ELECTRONIC SUPPLEMENTARY MATERIAL

Supplemental Table 1. Distribution of Survey Responses by Country

Country	Count (Percent)
Czechia	34 (23.1%)
France	13 (8.8%)
United Kingdom	11 (7.5%)
Hungary	10 (6.8%)
Germany	9 (6.1%)
Portugal	9 (6.1%)
Italy	8 (5.4%)
Poland	8 (5.4%)
Belgium	6 (4.1%)
Austria	4 (2.7%)
Greece	4 (2.7%)
Switzerland	4 (2.7%)
Croatia	3 (2.0%)
Estonia	3 (2.0%)
Latvia	3 (2.0%)
Slovenia	3 (2.0%)
Spain	3 (2.0%)
Other	12 (8.2%)

Supplemental Table 2. Radiologist-reported regulatory frameworks for LCS Reporting.

Country	National	Regional	Research Pilot	Not Regulated
Czechia	**24**	**2**	**10**	**0**
France	**1**	**1**	**11**	**4**
United Kingdom	**8**	**5**	**1**	**1**
Hungary	**2**	**2**	**5**	**1**
Germany	**4**	**0**	**2**	**3**
Portugal	**0**	**0**	**1**	**9**
Italy	**1**	**2**	**3**	**4**
Poland	**2**	**3**	**2**	**1**
Belgium	**1**	**1**	**2**	**3**
Austria	**1**	**1**	**1**	**4**
Greece	**0**	**0**	**0**	**4**
Switzerland	**0**	**0**	**2**	**2**
Croatia	**3**	**0**	**0**	**0**
Estonia	**0**	**0**	**3**	**0**
Latvia	**0**	**0**	**1**	**2**
Slovenia	**1**	**1**	**1**	**1**
Spain	**0**	**0**	**2**	**1**
Ireland	**1**	**0**	**1**	**0**
Luxembourg	**0**	**0**	**2**	**0**
Netherlands	**2**	**0**	**0**	**0**
Slovakia	**0**	**1**	**1**	**0**
Bulgaria	**0**	**0**	**1**	**1**
Serbia	**0**	**1**	**0**	**0**
Sweden	**0**	**0**	**1**	**0**
Turkey	**0**	**0**	**0**	**1**

Supplemental Table 3. Radiologist-reported regulatory frameworks for IF Reporting.

Country	National	Regional	Research Pilot	Not Regulated
Czechia	**24**	**0**	**0**	**0**
France	**0**	**0**	**10**	**5**
United Kingdom	**7**	**5**	**0**	**2**
Hungary	**2**	**1**	**3**	**4**
Germany	**2**	**0**	**1**	**6**
Portugal	**0**	**0**	**0**	**9**
Italy	**1**	**0**	**3**	**5**
Poland	**1**	**3**	**0**	**4**
Belgium	**1**	**1**	**2**	**3**
Austria	**1**	**1**	**1**	**4**
Greece	**0**	**0**	**0**	**4**
Switzerland	**0**	**0**	**2**	**2**
Croatia	**3**	**0**	**0**	**0**
Estonia	**0**	**0**	**2**	**1**
Latvia	**0**	**0**	**0**	**3**
Slovenia	**1**	**1**	**1**	**1**
Spain	**0**	**0**	**2**	**1**
Ireland	**1**	**0**	**1**	**0**
Luxembourg	**0**	**0**	**0**	**2**
Netherlands	**1**	**0**	**0**	**1**
Slovakia	**0**	**1**	**1**	**0**
Bulgaria	**0**	**0**	**1**	**0**
Serbia	**0**	**1**	**0**	**0**
Sweden	**0**	**0**	**1**	**0**
Turkey	**0**	**0**	**0**	**1**

Supplemental Figure S1: Management fo Incidental Findings Detected in Lung Cancer Screening – Survey

Management of Incidental Findings Detected in Lung Cancer Screening – Survey

1. **Are you a radiologist or a professional working in radiology?***
   a. Yes (Go to Q2)
   b. No (Message below displayed.)
   Thank you for your interest in contributing to our survey!
   At this time, we are specifically seeking input from radiology professionals.
   We highly encourage you to join the SOLACE Stakeholder Forum (europeanlung.org/lung). By joining, you'll receive updates and access to future surveys, including opportunities to share perspectives on incidental findings from a broader range of professionals.
   We deeply appreciate your continued interest and support in advancing the goals of the SOLACE Project!
2. **For which country do you conduct the majority of your lung cancer screening reporting?**
   a. Austria
   b. Belgium
   c. Bulgaria
   d. Croatia
   e. Republic of Cyprus
   f. Czech Republic
   g. Denmark
   h. Estonia
   i. Finland
   j. France
   k. Germany
   l. Greece
   m. Hungary
   n. Ireland
   o. Italy
   p. Latvia
   q. Lithuania
   r. Luxembourg
   s. Malta
   t. Netherlands
   u. Poland
   v. Portugal
   w. Romania
   x. Slovakia
   y. Slovenia
   z. Spain
   aa. Sweden
   bb. United Kingdom
   cc. Other (Go to Q3)
   A-BB (Go to Q4)
3. **Please specify other here.** (short answer)
4. **Please specify the types of screening programs in [Q2 Response] you are involved in:** (Check all that apply)
   a. National (e.g. EU member state level)
   b. Research/Implementation pilot (e.g. state/regional programmes or hospital level)
   c. Not within a programme (e.g. opportunistic screening)
   d. Other
5. **At which level(s) is lung cancer screening reporting regulated in [Q2 Response]?**
   a. National (e.g. EU member state level)
   b. Regional (e.g. state or regional legislation)
   c. Research pilot (e.g. study protocol)
   d. Not regulated
6. **At which level(s) is incidental finding as part of lung cancer screening reporting regulated in [Q2 Response]?**
   a. National (e.g. EU member state level) (Go to Q7)
   b. Regional (e.g. state or regional legislation) (Go to Q7)
   c. Research pilot (e.g. study protocol) (Go to Q7)
   d. Not regulated (Go to Q8)
7. **Indicate how each condition is REGULATED in the reporting of incidental findings:**
   a. Coronary artery calcification
      i. Mandatory/Always (Independent of Severity)
      ii. In Some Cases (Dependent on Severity)
      iii. Never
      iv. Not regulated
   b. Interstitial lung abnormalities
      i. Mandatory/Always (Independent of Severity)
      ii. In Some Cases (Dependent on Severity)
      iii. Never
      iv. Not regulated

c. Emphysema
    i. Mandatory/Always (Independent of Severity)
    ii. In Some Cases (Dependent on Severity)
    iii. Never
    iv. Not regulated
d. Bronchiectasis
    i. Mandatory/Always (Independent of Severity)
    ii. In Some Cases (Dependent on Severity)
    iii. Never
    iv. Not regulated
e. Consolidation and signs of infection
    i. Mandatory/Always (Independent of Severity)
    ii. In Some Cases (Dependent on Severity)
    iii. Never
    iv. Not regulated
f. Bronchial wall thickening
    i. Mandatory/Always (Independent of Severity)
    ii. In Some Cases (Dependent on Severity)
    iii. Never
    iv. Not regulated
g. Mediastinal lymph nodes
    i. Mandatory/Always (Independent of Severity)
    ii. In Some Cases (Dependent on Severity)
    iii. Never
    iv. Not regulated
h. Thyroid abnormalities
    i. Mandatory/Always (Independent of Severity)
    ii. In Some Cases (Dependent on Severity)
    iii. Never
    iv. Not regulated
i. Mediastinal mass
    i. Mandatory/Always (Independent of Severity)
    ii. In Some Cases (Dependent on Severity)
    iii. Never
    iv. Not regulated
j. Pleural effusions
    i. Mandatory/Always (Independent of Severity)
    ii. In Some Cases (Dependent on Severity)
    iii. Never
    iv. Not regulated
k. Pneumothorax and pneumomediastinum
    i. Mandatory/Always (Independent of Severity)
    ii. In Some Cases (Dependent on Severity)
    iii. Never
    iv. Not regulated
l. Diaphragm abnormalities
    i. Mandatory/Always (Independent of Severity)
    ii. In Some Cases (Dependent on Severity)
    iii. Never
    iv. Not regulated
m. Aortic aneurysms
    i. Mandatory/Always (Independent of Severity)
    ii. In Some Cases (Dependent on Severity)
    iii. Never
    iv. Not regulated
n. Breast nodules
    i. Mandatory/Always (Independent of Severity)
    ii. In Some Cases (Dependent on Severity)
    iii. Never
    iv. Not regulated
o. Liver lesions
    i. Mandatory/Always (Independent of Severity)
    ii. In Some Cases (Dependent on Severity)
    iii. Never
    iv. Not regulated
p. Renal lesions
    i. Mandatory/Always (Independent of Severity)
    ii. In Some Cases (Dependent on Severity)
    iii. Never
    iv. Not regulated

q. Bone abnormalities
    i. Mandatory/Always (Independent of Severity)
    ii. In Some Cases (Dependent on Severity)
    iii. Never
    iv. Not regulated
r. Adrenal lesions
    i. Mandatory/Always (Independent of Severity)
    ii. In Some Cases (Dependent on Severity)
    iii. Never
    iv. Not regulated
s. Respiratory bronchiolitis
    i. Mandatory/Always (Independent of Severity)
    ii. In Some Cases (Dependent on Severity)
    iii. Never
    iv. Not regulated
t. Pleural lesion with and without effusion
    i. Mandatory/Always (Independent of Severity)
    ii. In Some Cases (Dependent on Severity)
    iii. Never
    iv. Not regulated

8. Indicate how much you agree or disagree with the following statement:
LDCT is not a reliable technique to assess solid organs outside of the mediastinum.

a. Strongly agree
b. Moderately agree
c. Neither agree or disagree
d. Moderately disagree
e. Strongly disagree

9. Indicate how each condition in your OPINION should be regulated in the reporting of incidental findings:

a. Coronary artery calcification
    i. Mandatory/Always (Independent of Severity)
    ii. In Some Cases (Dependent on Severity) → Continue to 12a
    iii. Never
b. Interstitial lung abnormalities
    i. Mandatory/Always (Independent of Severity)
    ii. In Some Cases (Dependent on Severity) → Continue to 12b
    iii. Never
c. Emphysema
    i. Mandatory/Always (Independent of Severity)
    ii. In Some Cases (Dependent on Severity) → Continue to 12c
    iii. Never
d. Bronchiectasis
    i. Mandatory/Always (Independent of Severity)
    ii. In Some Cases (Dependent on Severity) → Continue to 12d
    iii. Never
e. Consolidation and signs of infection
    i. Mandatory/Always (Independent of Severity)
    ii. In Some Cases (Dependent on Severity) → Continue to 12e
    iii. Never
f. Bronchial wall thickening
    i. Mandatory/Always (Independent of Severity)
    ii. In Some Cases (Dependent on Severity) → Continue to 12f
    iii. Never
g. Mediastinal lymph nodes
    i. Mandatory/Always (Independent of Severity)
    ii. In Some Cases (Dependent on Severity) → Continue to 12g
    iii. Never
h. Thyroid abnormalities
    i. Mandatory/Always (Independent of Severity)
    ii. In Some Cases (Dependent on Severity) → Continue to 12h
    iii. Never
i. Mediastinal mass
    i. Mandatory/Always (Independent of Severity)
    ii. In Some Cases (Dependent on Severity) → Continue to 12i
    iii. Never
j. Pleural effusions
    i. Mandatory/Always (Independent of Severity)
    ii. In Some Cases (Dependent on Severity) → Continue to 12j
    iii. Never

k. Pneumothorax and pneumomediastinum
    i. Mandatory/Always (Independent of Severity)
    ii. In Some Cases (Dependent on Severity) → Continue to 12k
    iii. Never
l. Diaphragm abnormalities
    i. Mandatory/Always (Independent of Severity)
    ii. In Some Cases (Dependent on Severity) → Continue to 12l
    iii. Never
m. Aortic aneurysms
    i. Mandatory/Always (Independent of Severity)
    ii. In Some Cases (Dependent on Severity) → Continue to 12m
    iii. Never
n. Breast nodules
    i. Mandatory/Always (Independent of Severity)
    ii. In Some Cases (Dependent on Severity) → Continue to 12n
    iii. Never
o. Liver lesions
    i. Mandatory/Always (Independent of Severity)
    ii. In Some Cases (Dependent on Severity) → Continue to 12o
    iii. Never
p. Renal lesions
    i. Mandatory/Always (Independent of Severity)
    ii. In Some Cases (Dependent on Severity) → Continue to 12p
    iii. Never
q. Bone abnormalities
    i. Mandatory/Always (Independent of Severity)
    ii. In Some Cases (Dependent on Severity) → Continue to 12q
    iii. Never
r. Adrenal lesions
    i. Mandatory/Always (Independent of Severity)
    ii. In Some Cases (Dependent on Severity) → Continue to 12r
    iii. Never
s. Respiratory bronchiolitis
    i. Mandatory/Always (Independent of Severity)
    ii. In Some Cases (Dependent on Severity) → Continue to 12s
    iii. Never
t. Pleural lesion with and without effusion
    i. Mandatory/Always (Independent of Severity)
    ii. In Some Cases (Dependent on Severity) → Continue to 12t
    iii. Never

10. Do you believe that there should be any other conditions added to this list?
a. Yes (Go to Q11)
b. No (Go to Q12)

11. Please indicate the additional condition(s) that you believe should be added to this list. (Free text.)

12. Indicate what degree of severity each condition in your OPINION should be regulated in the reporting of incidental findings:
a. Coronary artery calcification
    i. High Severity
    ii. Moderate and High Severity
    iii. Low, Moderate, and High Severity
b. Interstitial lung abnormalities
    i. High Severity
    ii. Moderate and High Severity
    iii. Low, Moderate, and High Severity
c. Emphysema
    i. High Severity
    ii. Moderate and High Severity
    iii. Low, Moderate, and High Severity
d. Bronchiectasis
    i. High Severity
    ii. Moderate and High Severity
    iii. Low, Moderate, and High Severity
e. Consolidation and signs of infection
    i. High Severity
    ii. Moderate and High Severity
    iii. Low, Moderate, and High Severity
f. Bronchial wall thickening
    i. High Severity
    ii. Moderate and High Severity
    iii. Low, Moderate, and High Severity
g. Mediastinal lymph nodes
    i. High Severity
    ii. Moderate and High Severity
    iii. Low, Moderate, and High Severity
h. Thyroid abnormalities
    i. High Severity
    ii. Moderate and High Severity
    iii. Low, Moderate, and High Severity

i. Mediastinal mass
    i. High Severity
    ii. Moderate and High Severity
    iii. Low, Moderate, and High Severity
j. Pleural effusions
    i. High Severity
    ii. Moderate and High Severity
    iii. Low, Moderate, and High Severity
k. Pneumothorax and pneumomediastinum
    i. High Severity
    ii. Moderate and High Severity
    iii. Low, Moderate, and High Severity
l. Diaphragm abnormalities
    i. High Severity
    ii. Moderate and High Severity
    iii. Low, Moderate, and High Severity
m. Aortic aneurysms
    i. High Severity
    ii. Moderate and High Severity
    iii. Low, Moderate, and High Severity
n. Breast nodules
    i. High Severity
    ii. Moderate and High Severity
    iii. Low, Moderate, and High Severity
o. Liver lesions
    i. High Severity
    ii. Moderate and High Severity
    iii. Low, Moderate, and High Severity
p. Renal lesions
    i. High Severity
    ii. Moderate and High Severity
    iii. Low, Moderate, and High Severity
q. Bone abnormalities
    i. High Severity
    ii. Moderate and High Severity
    iii. Low, Moderate, and High Severity
r. Adrenal lesions
    i. High Severity
    ii. Moderate and High Severity
    iii. Low, Moderate, and High Severity
s. Respiratory bronchiolitis
    i. High Severity
    ii. Moderate and High Severity
    iii. Low, Moderate, and High Severity
t. Pleural lesion with and without effusion
    i. High Severity
    ii. Moderate and High Severity
    iii. Low, Moderate, and High Severity
